# Inflammatory Response Indices in Patients with Acute Ischemic Stroke Treated with and Without Reperfusion Therapy

**DOI:** 10.3390/jcm15010055

**Published:** 2025-12-21

**Authors:** Milena Świtońska, Agnieszka Rogalska, Natalia Mysiak, Agata Staniewska, Alicja Szulc, Oliwia Jarosz, Magdalena Konieczna-Brazis, Magdalena Grigorief, Daria Frąckowska, Jacek Budzyński

**Affiliations:** 1 Department of Neurology and Clinical Neurophysiology, Ludwik Rydygier Collegium Medicum in Bydgoszcz, Nicolaus Copernicus University in Toruń, No 2, 85-168 Bydgoszcz, Poland; m.switonska@cm.umk.pl (M.Ś.); alicja.szulc@cm.umk.pl (A.S.); magda.brazis@cm.umk.pl (M.K.-B.); 2 Jan Biziel University Hospital No. 2 in Bydgoszcz, Ludwik Rydygier Collegium Medicum in Bydgoszcz, Nicolaus Copernicus University in Toruń, 85-168 Bydgoszcz, Poland; agnieszka.rogalska@biziel.pl (A.R.); 503585@doktorant.umk.pl (N.M.); agata.staniewska@cm.umk.pl (A.S.); magdalena.grigorief@cm.umk.pl (M.G.); daria.frackowska@doktorant.umk.pl (D.F.); 3 Doctoral School of Medical and Health Sciences, Ludwik Rydygier Collegium Medicum in Bydgoszcz, Nicolaus Copernicus University in Toruń, 85-067 Bydgoszcz, Poland; 503671@doktorant.umk.pl; 4 Department of Vascular and Internal Medicine, Ludwik Rydygier Collegium Medicum in Bydgoszcz, Nicolaus Copernicus University in Toruń, 85-067 Bydgoszcz, Poland

**Keywords:** ischemic stroke, mechanical thrombectomy, reperfusion injury, inflammatory response

## Abstract

**Background:** Ischemic stroke remains a leading cause of mortality and long-term disability worldwide. Reperfusion therapies, such as intravenous thrombolysis and mechanical thrombectomy, are crucial for restoring cerebral blood flow but may also trigger ischemia–reperfusion injury and systemic inflammatory activation, associated with poorer clinical outcomes. **Methods:** We retrospectively analyzed medical records of 8833 patients hospitalized for acute ischemic stroke between January 2014 and May 2025. Of these, 2242 (25.38%) underwent reperfusion therapy (mechanical thrombectomy ± intravenous thrombolysis), and 6591 (74.62%) were treated conservatively. Laboratory parameters, including leukocyte count, C-reactive protein (CRP), and albumin, and composite inflammatory indices (e.g., neutrophil-to-lymphocyte ratio (NLR), systemic immune–inflammation index (SII), systemic-inflammation response index (SIRI), and neutrophil percentage-to-albumin ratio (NPAR)), were assessed at admission. Clinical outcomes included in-hospital mortality and functional scale results (e.g., National Institutes of Health Stroke Scale, modified Rankin score (mRS), Barthel scale, and Glasgow Coma Scale (GCS)). **Results:** Patients treated with reperfusion therapy had higher inflammatory indices (white blood cells, CRP, NLR, SII, and NPAR) compared to patients treated conservatively. In multiple regression analysis, these indices were significantly determined only by GCS and mRS scores, but age, gender, comorbidities, biochemical determinations, and type of ischemic stroke treatment (reperfusion or conservative) remained non-statistically significant. **Conclusions:** Patients with acute ischemic stroke undergoing reperfusion therapy exhibited a stronger inflammatory response and higher in-hospital mortality than those treated conservatively. However, multivariate analysis showed that a stronger inflammatory response following reperfusion therapy results more from the severity of the patients’ state than the kind of therapy.

## 1. Introduction

Acute ischemic stroke (AIS) remains one of the foremost causes of death and sustained disability worldwide, with a growing global burden associated with population aging and the persistence of cardiovascular risk factors, such as hypertension, diabetes mellitus, and atrial fibrillation [1,2]. The initial phase of AIS triggers a cascade of local and systemic inflammatory processes resulting from ischemia and reperfusion injury, oxidative stress, immune activation, and the release of enzymes (e.g., matrix metalloproteinase-9) [3,4,5,6,7,8,9,10,11]. These mechanisms, which include cytokine production, endothelial dysfunction, and leukocyte infiltration into ischemic cerebral tissue, contribute to neuronal damage, blood–brain barrier breakdown, and hemorrhagic stroke transformation, which may contribute to greater neurological deficit and mortality in the course of AIS [3,4,5]. Paradoxically, reperfusion therapy with intravenous thrombolysis and/or mechanical thrombectomy, which has been proven to be more effective than conservative treatment of AIS, may also, after cerebral blood restoration, induce ischemia–reperfusion processes, and secondary oxidoreductive stress may aggravate brain injury through cytokine production, augmentation of inflammatory cell migration, and changes in the balance of local and systemic inflammatory processes [3,4,5,6,7,8,9,10,11,12,13,14,15,16,17,18,19,20,21,22,23,24,25,26,27,28,29,30]. Locally, in response to signals released by platelets, neutrophils migrate to the site of the brain infarct within 6 to 24 h after brain ischemia. Next, neutrophils create neutrophil extracellular traps, secrete a number of cytokines and reactive oxygen and nitrogen species, and release inflammatory mediators and proteolytic enzymes, which amplify ischemic brain injury [3,31]. In the next phase, monocytes migrate to the site of the stroke. At the end of an inflammatory response process, 3–6 days after cerebral ischemia, lymphocytes accumulate in the brain. They exert a local regulatory and neuroprotective function through, inter alia, the secretion of interleukin-10, which may reduce final brain infarct volume and disability. These processes are associated with lymphopenia, reflecting the effect of stress-induced hypercortisolemia and systemic immunosuppression, which may predispose the patient to hospital-acquired infection (HAI).

The activity and sequences of respective white blood cells migration processes can be monitored using several markers, such as leukocyte and/or neutrophil count, platelet-to-lymphocyte ratio, and neutrophil-to-lymphocyte ratio, reflecting neutrophil exaltation and lymphocyte depletion [32,33]. Among the more complex systemic response markers, the most frequently used are the systemic immune–inflammation index (SII), systemic-inflammation response index (SIRI), and hemoglobin, albumin, lymphocyte, and platelet (HALP) score [14,24,25,26,27,28,29,30,31,34]. Higher values of these indices are associated with poor outcomes among patients with acute stroke, cardiovascular disease, and various types of cancer [4,10,14,16,35]. For example, in patients with AIS, these indices may determine futile recanalization after mechanical thrombectomy, final brain necrosis volume, and the severity of functional defect measured on various functional scales (e.g., National Institutes of Health Stroke Scale (NIHSS), Modified Rankin Scale (mRS), and Barthel and Norton scales). A higher intensity of inflammatory response to brain reperfusion injury may also, in a vicious circle pathomechanism, be the cause of the following: atherosclerosis plaque instability in the cerebral and other vascular beds (e.g., coronary and peripheral arteries), occurrence of major adverse cardiovascular events (MACE), myocardial infarction (MI), acute limb ischemia, additional stroke, hemorrhagic stroke transformation, atrial fibrillation, occurrence of respective subgroups of HAIs (e.g., pneumonia or urinary tract infections due to post-stroke systemic immunosuppression), prolongation of in-hospital stay (LOS), and nutritional complications due to the escalation of catabolic processes leading, for example, to pressure wounds, and, finally, higher neurological deficit or in-hospital death [4,10,14,16,34,35,36,37,38,39,40,41].

With regard to the data provided above, initiating therapy to control the activity of immune-inflammatory processes in the brain during AIS-related processes seems rational. Anti-inflammatory therapies now available, such as aspirin, canakinumab, and colchicine [25,28,42,43,44,45,46,47,48,49,50], might potentially control the inflammatory response to cerebral ischemia and improve the outcome of patients with AIS. These inflammatory-control agents seem to be the most useful for patients in whom reperfusion therapy was applied due to a dual source of inflammatory process induction and acceleration (ischemia and ischemia–reperfusion processes) [25]. However, before starting experimental anti-inflammatory therapy for AIS, it seems worth determining the biomarkers of the severity of inflammatory response to a cerebrovascular event and the kind of stroke treatment applied (reperfusion or conventional therapy), as well as their association with the poor outcome of the cerebrovascular event (in-hospital death, severity of neurological deficit, etc.). These biomarkers could be an additional criterion for patients’ qualification for reperfusion therapy for AIS (i.e., intravenous thrombolysis, mechanical thrombectomy), being helpful in avoiding the qualification of patients threatened with excessive inflammatory response and poor treatment outcomes.

The aim of this study is to compare the values of laboratory indices of inflammatory response between patients with AIS given reperfusion therapy (i.e., mechanical thrombectomy with or without preceding intravenous thrombolysis) and those treated conservatively.

## 2. Patients and Methods

### 2.1. Patients

We analyzed medical documentation of 8833 consecutive patients with AIS who were hospitalized in the Neurology Department of a university hospital due to acute ischemic stroke between 1 January 2014 and 31 May 2025. Of these patients, 2242 (25.38%) were treated with the intention of restoring cerebral blood flow (reperfusion therapy with endovascular mechanical thrombectomy (EMT) preceded in 1201 patients by intravenous thrombolysis (IVT)), and 6591 (74.62%) patients were treated conservatively. The inclusion criteria were: AIS (I63, according to the International Classification of Diseases, Tenth Revision, ICD-10) treated with EMT with or without preceding IVT with a recombinant tissue plasminogen activator (rtPA) (alteplase), given as causal (reperfusion) therapy; no history of leukemia; and being 18 or more years of age. The exclusion criteria were: hemorrhagic stroke, transient ischemic attack, or clinical evidence of infection or cancer at admission. The diagnosis of acute stroke was made by an experienced neurologist on duty and confirmed using computed tomography angiography as one criterion for qualification for EMT. Computed tomography and magnetic resonance imaging (MRI) of the brain were performed for all the patients enrolled. Comorbidities (hypertension, atrial fibrillation, diabetes mellitus, coronary artery disease, and peripheral artery disease, including carotid artery stenosis and chronic cardiac failure) were identified on the basis of the ICD-10 codes in the medical documentation. Endovascular mechanical thrombectomy was performed by an experienced, certificated interventional radiologist in patients with large-artery occlusion in the anterior circulation (in the majority, concerning the middle cerebral artery). Additional pharmacotherapy, physiotherapy, and patient management were applied in accordance with the criteria set out in the Polish Neurological Association Guideline.

### 2.2. Methods

Retrospective analysis was carried out on the medical documentation of all consecutive patients hospitalized in the Neurology Department of the university hospital due to AIS (I63, according to ICD-10) between 1 January 2014 and 31 May 2025. The following clinical and laboratory data were obtained, and measured using standard methods at the central hospital laboratory (the first measurement was made during hospitalization after admission to the Neurology Department and endovascular treatment, if it was performed): blood morphology from a blood smear examination of white blood cell (WBC, leukocytes); blood C-reactive protein (CRP); albumin; thyroid stimulating hormone (TSH); total, high-density lipoprotein (HDL), and low-density lipoprotein (LDL) cholesterol; triglyceride; glucose; HbA1c; and creatinine concentrations. Unfortunately, not all biochemical determinations mentioned were available for all patients.

The following single parameters were used as biomarkers of inflammatory response to AIS: blood CRP, interleukin-6, and albumin concentrations; WBC count; neutrophils, lymphocytes, and monocytes, both as counts and percentages of WBC count; and platelet count. We also calculated composite inflammatory response indices, such as: CRP-to-albumin ratio, CRP-to-lymphocyte ratio (CLR), neutrophil-to-lymphocyte ratio (NLR), neutrophil-to-platelet ratio (NPR), and lymphocyte-to-monocyte ratio (LMR); platelet-to-lymphocyte ratio (PLR), platelet count-to-albumin ratio (PAR), and lymphocyte-to-albumin ratio (LAR). Lastly, the following were calculated: Lymphocytes, albumin, and neutrophils (HLAN) index calculated according to the formula [hemoglobin (g/L) × lymphocytes (G/L) × albumin (g/L)/neutrophils (G/L)/100]; HALP index calculated as [hemoglobin (g/L) × albumin (g/L) × lymphocytes (G/L)/platelets (G/L)]; systemic immune–inflammation index calculated as [SII = neutrophil count × PLR]; systemic-inflammation response index, calculated according to the formula [SIRI = neutrophils × monocytes/lymphocytes); and the Naples prognostic score (NPS), calculated according to scored cut-offs for albumin, total cholesterol, NLR, and LMR.

We also calculated known nutritional risk indices, such as: Nutrition Risk Screening 2002 (NRS-2002) score; Nutritional Risk Index (NRI = [1.519 × serum albumin (g/L)] + [41.7 × weight (kg)/ideal body weight (kg)]; Geriatric Nutritional Risk Index (GNRI = [1.489 × albumin (g/L)] + [41.7 × current weight (kg)/ideal body weight (kg)]); and Controlling Nutritional Status (CONUT) score and class. The following values were accepted as correct, along with their interpretations for GNRI: GNRI > 98 = no risk of complications; GNRI 92-98 = low risk of complications; GNRI 82-92 = moderate risk of complications; and GNRI < 82 = major risk of complications. Ideal body weight (IBW) is calculated according to the Lorentz formula as follows: for women—IBW (kg) = height (cm) − 100 − {(height [cm] − 150)/2.5}; for men—IBW (kg) = height (cm) − 100 − {(height [cm] − 150)/4} [51].

Patients’ functional status and neurological deficits were determined according to NIHSS and mRS scales, as well as using the Glasgow Coma Scale (GCS), Barthel scale, Norton scale, Vulnerable Elders Survey (VES-13) score, and Morse Fall Scale (MFS).

### 2.3. Outcomes Measured

The following outcomes were measured and used in the analysis:

- Values of the single and composite inflammatory response indices referred to above;
- In-hospital all-cause mortality, readmission within 14, 30, and 365 days after discharge, length of in-hospital stay (LOS), changes (delta) in patient functional status (determined using NIHSS and mRS scores) between discharge and admission;
- The other scores of patients’ functional status assessment: GCS, Barthel scale, Norton scale, VES-13, and MFS.

### 2.4. Bioethics

The investigation was conducted in compliance with the Declaration of Helsinki for medical research with the permission of the local Bioethical Committee (No. KB 376/2025) on 25 June 2025.

### 2.5. Statistics

Statistical analysis was conducted using the licensed version of the statistical software Statistica, version 13.3, developed by Tibco Software, Inc. (2017), Palo Alto, California, San Ramon, CA 94583 United States. The normal distribution of the study variables was checked using the Kolmogorov–Smirnov test. Results are presented as the mean ± standard deviation, n, %, median, and interquartile range. The statistical significance of differences between groups was verified using Student’s *t*-test and the Chi^2^ test. Pearson or Spearman correlations were determined. The statistical significance level was set at a *p*-value of < 0.05. The optimal cut-offs for the parameters examined in predicting the occurrence of the respective outcomes were determined for maximal Youden indices by plotting receiver operating characteristic (ROC) curves. An area under the curve (AUC) with a 95% confidence interval (CI) was determined. A multivariate regression model was determined for each inflammatory response index mentioned above. The following were included as independent variables: patients’ age, gender, history of comorbidities (diabetes mellitus, atrial fibrillation, hypertension, chronic coronary syndrome, chronic kidney disease), performance of mechanical thrombectomy, body mass index (BMI), scores of GCS, mRS, NRS-2002, and blood concentrations of hemoglobin, LDL cholesterol, HbA1c, and creatinine. Logistic regression was also used.

## 3. Results

When compared to patients treated conservatively (n = 6591, 74.62%), patients with AIS who underwent reperfusion treatment (EMT ± IVT) (n = 2242, 25.38%) were statistically significantly older, had higher scores for an NRS-2002 survey, VES-3, and MFS at admission, as well as in mRS at admission and at discharge (Table 1). Moreover, in patients who underwent reperfusion therapy, we observed a shorter length of in-hospital stay, significantly lower scores in GCS, Barthel, and Norton scales at admission, as well as lower left ventricle ejection fraction in echocardiography (Table 1). Ischemic stroke patients treated with mechanical thrombectomy had a higher prevalence of all-cause in-hospital death and were less likely to be readmitted within 14, 30, and 365 days after discharge than patients treated conservatively (Table 1).

Of the biochemical determinations, compared to patients treated conservatively, those who underwent EMT had higher WBC and neutrophil counts, as well as higher blood creatinine and CRP. Moreover, patients on reperfusion therapy had lower platelet counts, a lower percentage of blood lymphocytes and eosinophils, and lower blood total, HDL, LDL, and non-HDL cholesterol, triglyceride, hemoglobin, HBA1c, and albumin concentrations (Table 2).

With regard to biomarkers of inflammatory response to a cerebrovascular event, compared to those who were treated conservatively, patients who underwent reperfusion treatment had higher values of ratios such as CRP-to-albumin, CRP-to-lymphocyte, CRP-to-monocyte, CRP-to-platelet, and neutrophil-to-lymphocyte counts; neutrophil-to-platelet and platelet-to-lymphocyte counts, and neutrophils to albumin; as well as for the composite indices (SII, SIRI, NPS, NRI, and neutrophil percentage-to-albumin ratio (NPAR)) (Table 3).

In a multivariate analysis using a multiple regression method, we tried to identify clinical and biochemical factors that determined independent variances of inflammatory response indices enumerated in Table 3 (data not presented in detail). We obtained statistically significant equations for CRP-to-albumin, -lymphocyte, -monocyte, and -platelet ratios with very low R^2^ coefficient values (0.06–0.10), in which the mRS score and blood hemoglobin concentration were the independent variables determining the variance of the indices mentioned. Whereas, in statistically significant equations for NLR, NPR, SII, SIRI, and neutrophil-to-albumin ratios, the GCS and mRS scores were the only independent variables determining variances of these indices, but with very low R^2^ coefficient values (0.09–0.15). Reperfusion treatment did not significantly influence any of the inflammatory response index measured.

In the final part of the assessment, we used ROC analysis to determine cut-off values for the respective inflammatory indices predictive of the occurrence of the measured outcomes (Table 4). We found that the highest, statistically significant AUCs in the prediction of all-cause in-hospital death were blood CRP concentration, neutrophil-to-albumin ratio, and neutrophil-to-lymphocyte ratio. In the prediction of early readmission within 14, 30, and 365 days, of the statistically significant cut-off values, CRP, WBC, neutrophil-to-albumin ratio, and neutrophil-to-lymphocyte ratio had the highest AUCs. However, only a few AUC values exceeded a threshold of 0.6, which indicated a low diagnostic value and scarce discriminative function of these inflammatory response indices [52]. None of the parameters studied had AUC values that surpassed a cut-off of 0.80. Nevertheless, for example, NLR > 4.59 was associated with significantly higher risk of all-cause in-hospital death in all patients (OR, 95%CI: 5.20; 4.26–6.31, *p* < 0.001), in patients treated with EMT (OR, 95%CI: 3.59; 2.69–4.87, *p* < 0.001), and in patients treated conservatively (OR, 95%CI: 4.80; 3.64–6.33, *p* < 0.001).

## 4. Discussion

In our study, we found that patients with AIS treated with reperfusion therapy had significantly higher values of the inflammatory response indices measured than subjects treated conservatively (Table 3). However, in multivariate analysis, EMT did not significantly influence any of the inflammatory response indices measured. We also found that patients treated with EMT had 2.8 times higher in-hospital mortality, lower risk of readmission, shorter LOS, and worse functional status than patients treated conservatively (Table 1). The outcomes measured were predicted by the inflammatory response indices analyzed, but with low diagnostic value and scarce discriminative function (Table 4).

The results we obtained corroborate reports by other authors showing relationships between greater values of inflammatory response indices and higher mortality, and lower probability of 90-day independence (mRS 0–2 scores) among patients with AIS [9,19,20,29,30,31,32,33,34,35,36,37,38,52,53]. Higher values of inflammatory response indices (mainly NLR) were also associated with the use of EMT, its outcome (blood flow restoration), and futile recanalization in the treatment of AIS in papers published by other authors [11,16,17,20,30,32,35]. For example, Li et al. reported elevated blood cytokine concentrations and neutrophil activation following EMT. These inflammatory response indices correlated with brain infarct size and the risk of hemorrhagic transformation [32]. Other authors also emphasized that post-stroke inflammation represents a critical determinant of unfavorable outcomes and that excessive immune activation may counteract the benefits of reperfusion (futile recanalization) [5,8,16,24,25,29,30,31,32,33,34,35,36,37,38,39,40,52,54,55]. The unfavorable associations revealed between higher in-hospital mortality and higher values of inflammatory response can be explained by a greater severity of ischemia–reperfusion processes rolling on in the brain and their aggravation by reperfusion therapy (i.e., EMT and/or IVT) [8,18].

Although several randomized controlled trials have confirmed improved reperfusion, earlier neurologic recovery, and better functional outcomes of EMT compared to IVT and conservative therapy for AIS [32], in our study, the in-hospital mortality and functional status (Table 1) in patients who underwent reperfusion therapy were worse than in patients treated conservatively. Our observations support the concept of a dual-edged role of reperfusion therapy. On the one hand, timely recanalization improves survival and functional independence; on the other hand, ischemia–reperfusion processes may worsen microvascular injury, disrupt the blood–brain barrier, and promote systemic inflammatory stress [3,4,5,6,7,8,9,10,11,12,13,14,15,16,17,18,19,20,21,22,23,24,25,26,27,28,29,30,31,32,33,34,35,36,37,38,39,40]. A more intense inflammatory response to acute cerebral ischemia and reperfusion was the only potential explanation for these poor outcomes (Table 3), although in our study, we did not confirm an influence of EMT on values of inflammatory response indices in multivariate analysis. On the other hand, low R^2^ coefficients suggested the importance of still unidentified factors in the modulation of inflammatory response to brain injury. In our study, we also found a lower number of readmissions within 14, 30, and 365 days after discharge (Table 1), which might be a substitute for better late prognosis in patients with AIS treated with EMT. However, we realize the limitation of such an assumption, because patients who have passed away cannot be readmitted. These doubts do not resolve the analysis of scores in numerous functional scales (mRS, MFS, GCS, Bathel, Norton, and VES-13), which showed a worse functional status for patients who underwent reperfusion therapy compared to patients treated conservatively (Table 1).

The association between elevated inflammatory indices and higher in-hospital all-cause mortality observed in our study, as well as an excessive immune-inflammatory response to brain ischemia as a main pathomechanism of brain injury deterioration after reperfusion therapy, highlights the clinical need to evaluate adjunctive anti-inflammatory or antioxidant therapies in AIS patients undergoing EMT with or without IVT in order to protect them against harmful ischemia–reperfusion processes evoked by cerebral blood flow restoration [24,25,42,46,47,48,56,57,58]. Pre-clinical and translational research has shown that agents, such as uric acid, edaravone, or selective cytokine inhibitors (e.g., canakinumab and colchicine), may mitigate reperfusion-related brain injury [24,25,42,43,44,45,46,47,48,56,57,58]. Ongoing clinical trials are exploring whether the modulation of post-stroke inflammation can improve neurological outcomes without increasing the risk of infection or bleeding [3,49].

As with the majority of studies, our analysis has some limitations that may reduce the strength of the conclusions drawn. The study design should be considered the main study limitation. Our analysis was performed in only one center and was a retrospective analysis of medical documentation. However, the study was performed on a large sample size containing almost 9000 subjects, with a 1:3 proportion of patients who underwent reperfusion or conservative treatment. Secondly, laboratory data that were the basis of comparisons drawn from Table 3 were not available for all the patients who underwent EMT during the period analyzed. Thirdly, we did not analyze serial measurements of the inflammatory response indices, and a single determination at admission to the clinic might be biased [55]. Nonetheless, it is known that respective types of white blood cells migrate from the blood to the focus of necrosis in the brain at different times after brain ischemia occurs: first neutrophils (6–24 h), next monocytes, and last lymphocytes (between 3 and 6 days) [8,32,39]. Fourthly, NIHSS scores at admission and discharge were determined only in patients who underwent EMT. Fifthly, we did not analyze the associations between values of inflammatory response indices measured and efficacy of EMT and restoration of vessel patency, the number of catheter passages (reperfusion procedures), and volume of ischemic brain tissue; these EMT-specific metrics will be addressed in future dedicated studies.

Future studies should also focus on integrating values of inflammatory biomarkers with neuroimaging measures of reperfusion injury (e.g., blood–brain barrier disruption on MRI) and clinical outcomes [18,22]. A prospective design, including serial blood sampling, may clarify whether specific inflammatory patterns predict hemorrhagic complications or unfavorable recovery [32,55]. Finally, interventional trials testing anti-inflammatory strategies in EMT-treated patients are warranted to determine whether modulation of post-stroke inflammation can improve survival and functional independence [12,25,27,40,47,48,55,56,57,58,59].

## 5. Conclusions

In our study, patients with AIS who underwent reperfusion therapy had higher values of inflammatory response indices and in-hospital all-cause mortality than patients treated conservatively. Although multivariate analysis did not confirm a significant influence of EMT on values of the inflammatory response indices measured, our study demonstrated the importance of still unidentified, individually different factors modulating severities of ischemia–reperfusion processes rolling in the brain during AIS. This highlights the necessity to plan further studies on the effectiveness and safety of anti-inflammatory agents or antioxidants in the prevention of the harmful effects of reperfusion therapy for AIS.

## Figures and Tables

**Table 1:** Clinical characteristics of patients with acute ischemic stroke undergoing reperfusion treatment and conservative therapy.

Parameter	Reperfusion Therapy (n = 2242; 25.38%)	Conservative Treatment (n= 6591; 74.62%)	*p*
Age (years)	71.94 ± 13.05	70.50 ± 12.57	<0.001
Male gender (n, %)	1079 (48.13%)	3211 (48.72%)	0.629
Diabetes mellitus (n, %	168 (7.49%)	541 (8.21%)	0.282
Hypertension (n, %)	527 (23.51%)	1768 (26.82%)	<0.01
Atrial fibrillation (n, %)	274 (12.22%)	508 (7.71%)	<0.001
Chronic cardiac failure according to echocardiography (LVEF ≤ 40%, LVEF 41–49%; LVEF ≥ 50%)- LVEF was determined in 4555 pts; 542 vs. 4013 pts in respective group	60 (11.07%) 21 (3.87%) 461 (85.06%)	171 (4.26%) 141 (3.51%) 3701 (92.23%)	<0.001
Length of in-hospital stay (days)	9.08 ± 11.67	10.80 ± 10.02	<0.001
Delay between emergency department admission and admission to neurological ward (hours)	1.01 ± 0.09	1.05 ± 0.23	<0.001
Intravenous thrombolysis preceding mechanical thrombectomy (n, %)	1201 (53.6%)	0	
All-cause in-hospital death (n, %)	484 (21.59%)	588 (8.92%)	<0.001
Readmission during 14 days after discharge (n, %)	43 (1.92%)	258 (3.91%)	<0.001
Readmission during 30 days after discharge (n, %)	91 (4.06%)	547 (8.30%)	<0.001
Readmission during 365 days after discharge (n, %)	274 (12.22%)	2129 (32.30%)	<0.001
BMI (kg/m^2^)	28.24 ± 6.10	28.00 ± 10.95	0.747
% of ideal body mass (%)	129.38 ± 28.25	124.13 ± 21.74	0.518
NRS 2002 score	2.52 ± 0.76	1.82 ± 1.14	<0.001
GCS score	12.50 ± 3.24	14.32 ± 1.92	<0.001
Barthel scale score	29.39 ± 37.61	67.14 ± 38.01	<0.001
NIHSS score at admission	14.49 ± 5.98		
NIHSS score at discharge	11.13 ± 7.28		
mRS score at admission	3.34 ± 1.60	1.96 ± 1.61	<0.001
mRS score at discharge	2.60 ± 1.68	1.61 ± 1.50	<0.001
Norton scale score	9.95 ± 3.27	15.19 ± 4.34	<0.001
VES-13 score	4.87 ± 3.39	4.69 ± 3.26	0.144
Score in 3rd question of VES-13	1.24 ± 0.94	1.35 ± 0.90	0.001
Score in 4th question of VES-13	2.25 ± 1.99	2.14 ± 2.00	0.143
MFS score	36.55 ± 17.11	27.65 ± 19.20	<0.001

Abbreviations: BMI—body mass index; GCS—Glasgow Coma Scale; MFS—Morse Fall Scale; NIHSS—National Institutes of Health Stroke Scale; NRS 2002—Nutritional Risk Screening 2002 mRS—modified Rankin Scale; VES-13—Vulnerable Elders Survey score.

**Table 2:** Biochemical parameters in patients with acute ischemic stroke who underwent reperfusion treatment vs. conservative therapy.

Parameter	Reperfusion Therapy (n = 2242; 25.38%)	Conservative Treatment (n= 6591; 74.62%)	*p*
Red blood cells (T/L)	4.26 ± 0.61	4.56 ± 1.35	<0.001
Hemoglobin (g/L)	12.97 ± 1.91	13.80 ± 1.74	<0.001
Hematocrit (%)	38.28 ± 5.28	40.73 ± 4.70	<0.001
White blood cells (WBC, G/L)	11.10 ± 5.11	9.16 ± 5.11	<0.001
Platelet count (G/L)	228.31 ± 80.26	239.73 ± 81.36	<0.001
Neutrophil count (G/L)	8.77 ± 4.48	6.70 ± 4.10	<0.001
Neutrophil percentage (%)	74.26 ± 12.55	67.64 ± 13.76	<0.001
Lymphocyte count (G/L)	1.86 ± 4.58	2.13 ± 4.79	0.130
Lymphocyte percentage (%)	16.28 ± 10.85	21.46 ± 11.68	<0.001
Monocyte count (G/L)	0.86 ± 0.49	0.82 ± 1.04	0.359
Eosinophil count (G/L)	1.94 ± 1.44	2.78 ± 4.89	0.061
Eosinophil percentage (%)	1.03 ± 1.54	1.71 ± 1.86	<0.001
Total cholesterol (mg/dL)	142.95 ± 40.80	154.09 ± 63.62	0.003
HDL cholesterol (mg/dL)	43.65 ± 14.68	49.53 ± 16.58	<0.001
Non-HDL cholesterol (mg/dL)	106.36 ± 42.08	108.61 ± 47.93	0.629
LDL cholesterol (mg/dL)	100.70 ± 44.23	109.68 ± 45.36	<0.001
Triglycerides (mg/dL)	117.79 ± 61.39	129.02 ± 118.28	0.049
Glucose (mg/dL)	137.21 ± 50.36	141.27 ± 60.07	0.093
HBA1c (%)	6.17 ± 1.51	6.67 ± 1.69	<0.001
Creatinine (mg/dL)	1.07 ± 2.56	1.00 ± 0.44	0.030
Albumin (g/L)	3.29 ± 0.51	3.46 ± 0.69	<0.001
CRP (mg/dL)	18.34 ± 33.75	11.41 ± 27.65	<0.001
aptt (s)	29.09 ± 12.79	29.77 ± 11.26	0.038
INR	1.11 ± 0.32	1.13 ± 1.42	0.767
Uric acid (mg/dL)	5.77 ± 2.09	5.86 ± 2.07	0.497
TSH (mU/L)	1.52 ± 2.06	2.13 ± 6.04	<0.001

Abbreviation: CRP—C-reactive protein; HDL—high-density lipoprotein; INR—international normalized ratio; LDL—low-density lipoprotein; TSH—thyrotropin-stimulating hormone.

**Table 3:** Inflammatory indices in patients with acute ischemic stroke undergoing reperfusion treatment vs. conservative therapy.

Parameter	Reperfusion Therapy (n= 2242; 25.38%)	Conservative Treatment (n= 6591; 74.62%)	*p*
CRP-to-albumin ratio	2.71; 0.78–7.37	1.29; 0.44–4.30	0.002
CRP-to-lymphocyte ratio	4.80; 1.47–14.93	1.83; 0.69–6.03	<0.001
CRP-to-neutrophil ratio	0.85; 0.33–2.21	0.54; 0.21–1.53	0.690
CRP-to-monocyte ratio	8.31; 3.19–24.00	4.36; 1.79–12.32	<0.001
CRP-to-platelet ratio	0.03; 0.01–0.08	0.01; 0.01–0.04	<0.001
Neutrophil-to-lymphocyte ratio (NLR)	5.44; 3.15–9.55	3.34; 2.05–6.10	<0.001
Neutrophil-to-platelet ratio (NPR)	52.10; 34.28–82.42	40.73; 26.76–65.33	<0.001
Platelet-to-lymphocyte ratio (PLR)	154.75; 105.26–220.95	138.64; 98.51–205.24	0.006
Platelet-to-albumin ratio	67.65; 51.19–89.81	65.79; 51.55–85.35	0.265
Lymphocyte-to-albumin ratio	0.40; 0.27–0.54	0.44; 0.29–0.60	0.160
Neutrophil-to-albumin ratio	2.71; 1.92–3.97	1.90; 1.20–3.02	<0.001
Lymphocyte-to-monocyte ratio (LMR)	1.87; 1.23–2.80	2.47; 1.59–3.47	0.504
HLAN	5.97; 3.32–10.35	10.00; 506–19.46	0.127
HALP	23.51; 15.00–36.89	28.42; 16.94–46.27	0.161
SII	1192.27; 659.22–2148.52	766.36; 445–1446.27	<0.001
SIRI	4.17; 2.11–8.16	2.21; 1.27–4.61	<0.001
NPS	4.00; 3.00–4.00	3.00; 3.00–4.00	<0.001

Annotation: data presented as median; IQR—interquartile range, U—Mann–Whitney test; CRP—C-reactive protein; HALP—hemoglobin, albumin, lymphocytes, platelets index; HLAN—hemoglobin, lymphocytes, albumin, neutrophils index; NPS—Naples prognostic score; SII—systemic immune–inflammation index; SIRI—systemic-inflammation response index.

**Table 4:** Inflammatory response indices as predictors of measured outcomes in ROC analysis.

Parameter	In-Hospital Death		14-Day Readmission		30-Day Readmission		365-Day Readmission	
	Cut-Off Value	AUC, 95%CI, *p*	Cut-Off Value	AUC, 95%CI, *p*	Cut-Off Value	AUC, 95%CI, *p*	Cut-Off Value	AUC, 95%CI, *p*
WBC	3.26	0.32; 0.30–0.34; <0.001	7.93	0.57; 0.54–0.60; <0.001	8.09	0.54, 0.51–0.56; 0.002	8.49	0.56, 0.55–0.57; <0.001
Neutrophils	0.10	0.25, 0.23–0.27; <0.001	5.54	0.56; 0.52–0.60; 0.006	5.57	0.56, 0.53–0.59; 0.001	6.41	0.59, 0.57–0.61; <0.001
CRP	7.0	0.55, 0.54–0.57; <0.001	0.80	0.50, 0.47–0.53; 0.978	23.00	0.50, 0.48–0.53; 0.924	7.0	0.56, 0.54–0.57; <0.001
Albumin	1.89	0.44, 0.42–0.47; <0.001	4.29	0.52; 0.47–0.79; 0.431	3.22	0.50, 0.46–0.54; 0.923	1.89	0.44, 0.42–0.47; <0.001
CRP-to-albumin ratio	1.13	0.51, 0.47–0.55; 0.762	16.20	0.43, 0.41–0.46; <0.001	9.97	0.50, 0.45–0.53; 0.751	1.11	0.57, 0.54–0.57; <0.001
CRP-to-lymphocyte ratio	1.09	0.50, 0.46–0.52; 0.702	4.40	0.56, 0.54–0.57; <0.001	1.09	0.49, 0.46–0.52; 0.702	4.40	0.56, 0.54–0.59; <0.001
Neutrophil-to-lymphocyte ratio	4.59	0.54, 0.51–0.57; 0.004	4.70	0.57, 0.57–0.59; <0.001	4.59	0.54, 0.51–0.57; 0.004	4.70	0.57; 0.56–0.59; <0.001
Neutrophil-to-platelet ratio	44.57	0.52, 0.49–0.55; 0.235	43.45	0.55, 0.53–0.56; <0.001	44.57	0.52, 0.49–0.55; 0.235	43.45	0.55; 0.53–0.56; <0.001
Platelet-to-lymphocyte ratio	202.11	0.49, 0.46–0.53; 0.390	142.42	0.52, 0.50–0.54; 0.048	202.11	0.49, 0.44–0.52; 0.390	142.42	0.52; 0.50–0.54; 0.048
Platelet-to-albumin ratio	154.55	0.47, 0.43–0.51; 0.142	76.65	0.50, 0.47–0.52; 0.841	154.55	0.47, 0.43–0.51; 0.141	76.65	0.49, 0.47–0.52; 0.842
Lymphocyte-to-albumin ratio	0.81	0.48, 0.43–0.53; 0.376	1.00	0.47, 0.43–0.50; 0.037	0.81	0.48, 0.43–0.53; 0.375	1.00	0.47, 0.43–0.50; 0.037
Neutrophil-to-albumin ratio	1.92	0.57, 0.52–0.61; 0.004	2.07	0.62, 0.59–0.65; <0.001	1.92	0.57, 0.52–0.61; 0.004	2.07	0.62; 0.59–0.65; <0.001
Lymphocyte-to-monocyte ratio	4.73	0.48; 0,45–0.51; 0.252	5.37	0.45, 0.43–0.47; <0.001	4.73	0.48, 0.55–0.51; 0.252	5.37	0.45, 0.43–0.47; <0.001
HLAN	38.17	0.45; 0.40–0.49; 0,024	442.88	0.40; 0.36–0.42, <0.001	38.17	0.45, 0.40–0.49; 0.024	443.88	0.39, 0.364–0.422; <0.001
HALP	32.86	0.51, 0.47–0.56; 0.567	94.42	0.46; 0,44–0.50; 0.033	32.86	0.51, 0.47–0.56; 0.567	94.42	0.46; 0.44–0.50; 0.033
SII	1.14	0.53, 0.50–0.56; 0.087	1038.73	0.56, 0.54–0.58; <0.001	504.91	0.53, 0.50–0.56; 0.087	1038.73	0.56; 0.54–0.58; <0.001
SIRI	3.07	0.54, 0.51–0.57; 0.006	2.93	0.57, 0.56–0.59; <0.001	3.07	0.54, 0.51–0.57; 0.006	2.93	0.57; 0.56–0.59; <0.001
NPS	4.00	0.56, 0.50–0.62; 0.057	4.00	0.603, 0.56–0.64; <0.001	4.00	0.56, 0.50–0.62; 0.057	4.0	0.60, 0.56–0.64; <0.001

Abbreviations: CRP—C-reactive protein; HALP—hemoglobin, albumin, lymphocytes, platelets index; HLAN—hemoglobin, lymphocytes, albumin, neutrophils index; NPS—Naples prognostic score; SII—systemic immune–inflammation index; SIRI—systemic-inflammation response index; WBC—white blood cells.

## Data Availability

The original contributions presented in this study are included in the article. Further inquiries can be directed to the corresponding author.

## References

[1] GBD 2019 Stroke Collaborators (2021). Global, regional, and national burden of stroke and its risk factors, 1990–2019: A systematic analysis for the Global Burden of Disease Study 2019. Lancet Neurol..

[2] Hasan T.F., Rabinstein A.A., Middlebrooks E.H., Haranhalli N., Silliman S.L., Meschia J.F., Tawk R.G. (2018). Diagnosis and Management of Acute Ischemic Stroke. Mayo Clin. Proc..

[3] Lambertsen K.L., Finsen B., Clausen B.H. (2019). Post-stroke inflammation-target or tool for therapy?. Acta Neuropathol..

[4] Lin K.B., Fan F.H., Cai M.Q., Yu Y., Fu C.L., Ding L.Y., Sun Y.D., Sun J.W., Shi Y.W., Dong Z.F. (2022). Systemic immune inflammation index and system inflammation response index are potential biomarkers of atrial fibrillation among the patients presenting with ischemic stroke. Eur. J. Med. Res..

[5] Jayaraj R.L., Azimullah S., Beiram R., Jalal F.Y., Rosenberg G.A. (2019). Neuroinflammation: Friend and foe for ischemic stroke. J. Neuroinflamm..

[6] Saver J.L., Goyal M., van der Lugt A., Menon B.K., Majoie C.B., Dippel D.W., Campbell B.C., Nogueira R.G., Demchuk A.M., Tomasello A. (2016). Time to Treatment With Endovascular Thrombectomy and Outcomes From Ischemic Stroke: A Meta-analysis. JAMA.

[7] Aierken K., Ma L., Zhu Y., Jin X., Zhu Y., Zhou J., Gao J., Zhao H., Wang T., Li S. (2025). The association between the systemic immune-inflammation index and in-hospital mortality among acute ischemic stroke with atrial fibrillation patients undergoing intravenous thrombolysis. Front. Cardiovasc. Med..

[8] Chen P.Y., Chen G.C., Hsiao C.L., Hsu P.J., Yang F.Y., Liu C.Y., Tsou A., Chang W.L., Liu H.H., Lin S.K. (2022). Comparison of Clinical Features, Immune-Inflammatory Markers, and Outcomes Between Patients with Acute In-Hospital and Out-of-Hospital Ischemic Stroke. J. Inflamm. Res..

[9] Cheng W., Zhao Q., Li C., Xu Y. (2023). Neuroinflammation and brain-peripheral interaction in ischemic stroke: A narrative review. Front. Immunol..

[10] Chu M., Luo Y., Wang D., Liu Y., Wang D., Wang Y., Zhao J. (2023). Systemic inflammation response index predicts 3-month outcome in patients with mild acute ischemic stroke receiving intravenous thrombolysis. Front. Neurol..

[11] Chu M., Wang D. (2024). The systemic inflammation score is a prognostic factor for patients with ischemic stroke who have not undergone intravenous thrombolysis or endovascular thrombectomy therapy. Clin. Neurol. Neurosurg..

[12] Burkard P., Vögtle T., Nieswandt B. (2020). Platelets in Thrombo-Inflammation: Concepts, Mechanisms, and Therapeutic Strategies for Ischemic Stroke. Hamostaseologie.

[13] Chen G., Wang A., Zhang X., Li Y., Xia X., Tian X., Li J., Miao Z., Yue W. (2024). Systemic Immune-Inflammation Response is Associated with Futile Recanalization After Endovascular Treatment. Neurocrit Care.

[14] Chen J., Hu R., Shang L., Li X., Lin Y., Yao Y., Hu C. (2024). The HALP (hemoglobin, albumin, lymphocyte, and platelet) score is associated with hemorrhagic transformation after intravenous thrombolysis in patients with acute ischemic stroke. Front. Neurol..

[15] Wang N., Wang L., Zhang M., Deng B., Wu T. (2024). Correlations of 2 Novel Inflammation Indexes With the Risk for Early Neurological Deterioration in Acute Ischemic Stroke Patients After Intravenous Thrombolytic Therapy. Neurologist.

[16] She J., Gao W., Zhao Y., Cai L., Ge H., Chen X., Chen Z., Zhu R. (2025). Dynamic changes of systemic immune-inflammation index and systemic inflammation response index after mechanical thrombectomy and their predictive value for functional outcomes in patients with acute ischemic stroke. Neurol. Asia.

[17] Lattanzi S., Norata D., Divani A.A., Di Napoli M., Broggi S., Rocchi C., Ortega- Gutierrez S., Mansueto G., Silvestrini M. (2021). Systemic Inflammatory Response Index and Futile Recanalization in Patients with Ischemic Stroke Undergoing Endovascular Treatment. Brain Sci..

[18] Gauberti M., Lapergue B., Martinez de Lizarrondo S., Vivien D., Richard S., Bracard S., Piotin M., Gory B. (2018). Ischemia-Reperfusion Injury After Endovascular Thrombectomy for Ischemic Stroke. Stroke.

[19] Kan Y., Yang L., Ren C., Li C., Xu J., Guo W., Zhao W., Ji X. (2025). The Effect of Systemic Inflammatory Response on Mechanical Thrombectomy is Partly Mediated by Pre-thrombectomy Cerebral Edema in Acute Stroke Patients. Curr. Neurovasc Res..

[20] Ferro D., Matias M., Neto J., Dias R., Moreira G., Petersen N., Azevedo E., Castro P. (2021). Neutrophil-to-Lymphocyte Ratio Predicts Cerebral Edema and Clinical Worsening Early After Reperfusion Therapy in Stroke. Stroke.

[21] Soldozy S., Dalzell C., Skaff A., Ali Y., Norat P., Yagmurlu K., Park M.S., Kalani M.Y.S. (2023). Reperfusion injury in acute ischemic stroke: Tackling the irony of revascularization. Clin. Neurol. Neurosurg..

[22] Mizuma A., Yenari M.A. (2017). Anti-Inflammatory Targets for the Treatment of Reperfusion Injury in Stroke. Front. Neurol..

[23] Mizuma A., You J.S., Yenari M.A. (2018). Targeting Reperfusion Injury in the Age of Mechanical Thrombectomy. Stroke.

[24] Yang L., Wang M. (2025). Association of Systemic Immune-Inflammation Index With Stroke and Mortality Rates: Evidence From the NHANES Database. Neurologist.

[25] Yang M., Liu B., Chen B., Shen Y., Liu G. (2025). Cerebral ischemia-reperfusion injury: Mechanisms and promising therapies. Front. Pharmacol..

[26] Wang D., Ma L., Li Z., Ye G., Chen M. (2025). Significant association between systemic immune-inflammation index and stroke. Medicine.

[27] Wang H., Zhang S., Xie L., Zhong Z., Yan F. (2023). Neuroinflammation and peripheral immunity: Focus on ischemic stroke. Int. Immunopharmacol..

[28] Wang N., Yang Y., Qiu B., Gao Y., Wang A., Xu Q., Meng X., Xu Y., Song B., Wang Y. (2022). Correlation of the systemic immune-inflammation index with short- and long-term prognosis after acute ischemic stroke. Aging.

[29] Wang S., Zhang L., Qi H., Zhang F.L., Fang Q., Qiu L. (2023). Pan-Immune-Inflammatory Value Predicts the 3 Months Outcome in Acute Ischemic Stroke Patients after Intravenous Thrombolysis. Curr. Neurovasc Res..

[30] Wei C.J., Xue J.J., Zhou X., Xia X.S., Li X. (2024). Systemic Immune-Inflammation Index is a Prognostic Predictor for Patients With Acute Ischemic Stroke Treated With Intravenous Thrombolysis. Neurologist.

[31] Weng Y., Zeng T., Huang H., Ren J., Wang J., Yang C., Pan W., Hu J., Sun F., Zhou X. (2021). Systemic Immune-Inflammation Index Predicts 3-Month Functional Outcome in Acute Ischemic Stroke Patients Treated with Intravenous Thrombolysis. Clin. Interv. Aging.

[32] Li S.J., Cao S.S., Huang P.S., Nie X., Fu Y., Liu J.R. (2022). Post-operative neutrophil-to-lymphocyte ratio and outcome after thrombectomy in acute ischemic stroke. Front. Neurol..

[33] Świtońska M., Piekuś-Słomka N., Słomka A., Sokal P., Żekanowska E., Lattanzi S. (2020). Neutrophil-to-Lymphocyte Ratio and Symptomatic Hemorrhagic Transformation in Ischemic Stroke Patients Undergoing Revascularization. Brain Sci..

[34] Ma F., Li L., Xu L., Wu J., Zhang A., Liao J., Chen J., Li Y., Li L., Chen Z. (2023). The relationship between systemic inflammation index, systemic immune-inflammatory index, and inflammatory prognostic index and 90-day outcomes in acute ischemic stroke patients treated with intravenous thrombolysis. J. Neuroinflamm..

[35] Huang S., Xie W., Gao Y., Jin Y., Chen Y., Zhou G., Chen F., Jin Q., Wu Z., Wang L. (2024). A Role for Systemic Inflammation in Stroke-Associated Infection and the Long-Term Prognosis of Acute Ischemic Stroke: A Mediation Analysis. J. Inflamm. Res..

[36] Zhou Y., Yang Q., Zhou Z., Yang X., Zheng D., He Z., Liu Y., Xu T., Yin Y., Wei W. (2025). Correction: Systemic immune-inflammation index is associated with clinical outcome of acute ischemic stroke patients after intravenous thrombolysis treatment. PLoS ONE.

[37] Hervella P., Pérez-Mato M., Rodríguez-Yáñez M., López-Dequidt I., Pumar J.M., Sobrino T., Campos F., Castillo J., da Silva-Candal A., Iglesias-Rey R. (2021). sTWEAK as Predictor of Stroke Recurrence in Ischemic Stroke Patients Treated With Reperfusion Therapies. Front. Neurol..

[38] Huang Y.W., Zhang Y., Feng C., An Y.H., Li Z.P., Yin X.S. (2023). Systemic inflammation response index as a clinical outcome evaluating tool and prognostic indicator for hospitalized stroke patients: A systematic review and meta-analysis. Eur. J. Med. Res..

[39] Kim J.Y., Park J., Chang J.Y., Kim S.H., Lee J.E. (2016). Inflammation after Ischemic Stroke: The Role of Leukocytes and Glial Cells. Exp. Neurobiol..

[40] Luo Y., Dong W., Yuan L., Zhu Y.A., Zhang D.D., Ni H., Zhu W. (2025). The Role of Thrombo- inflammation in Ischemic Stroke: Focus on the Manipulation and Clinical Application. Mol. Neurobiol..

[41] Qian F., Du X., He Y. (2025). Causal association of inflammation with ischemic stroke and its subtypes: A bidirectional Mendelian randomization study. J. Stroke Cerebrovasc. Dis..

[42] Wang Y., Li J., Johnston S.C., Hankey G.J., Easton J.D., Meng X., Shi F.D., Wang Y., Zhao X., Li Z. (2023). Colchicine in High-risk Patients with Acute Minor-to-moderate Ischemic Stroke or Transient Ischemic Attack (CHANCE-3): Rationale and design of a multicenter randomized placebo-controlled trial. Int. J. Stroke.

[43] Shaji N., Storey R.F., Parker W.A.E. (2023). Drug therapies for stroke prevention. Br. J. Cardiol..

[44] Chaturvedi S., De Marchis G.M. (2024). Inflammatory Biomarkers and Stroke Subtype: An Important New Frontier. Neurology.

[45] Goh C.X.Y., Tan Y.K., Tan C.H., Leow A.S.T., Ho J.S.Y., Tan N.H.W., Goh S., Ho A.F.W., Sharma V.K., Chan B.P.L. (2022). The use of colchicine as an anti-inflammatory agent for stroke prevention in patients with coronary artery disease: A systematic review and meta-analysis. J. Thromb. Thrombolysis.

[46] Ren Y., Wei B., Song X., An N., Zhou Y., Jin X., Zhang Y. (2015). Edaravone’s free radical scavenging mechanisms of neuroprotection against cerebral ischemia: Review of the literature. Int. J. Neurosci..

[47] Pérez-Mato M., López-Arias E., Bugallo-Casal A., Correa-Paz C., Arias S., Rodríguez-Yáñez M., Santamaría-Cadavid M., Campos F. (2024). New Perspectives in Neuroprotection for Ischemic Stroke. Neuroscience.

[48] Dammavalam V., Lin S., Nessa S., Daksla N., Stefanowski K., Costa A., Bergese S. (2024). Neuroprotection during Thrombectomy for Acute Ischemic Stroke: A Review of Future Therapies. Int. J. Mol. Sci..

[49] Tack R.W.P., Amboni C., van Nuijs D., Pekna M., Vergouwen M.D.I., Rinkel G.J.E., Hol E.M. (2025). Inflammation, Anti-inflammatory Interventions, and Post-stroke Cognitive Impairment: A Systematic Review and Meta-analysis of Human and Animal Studies. Transl. Stroke Res..

[50] Budzyński J., Tojek K., Czerniak B., Banaszkiewicz Z. (2016). Scores of nutritional risk and parameters of nutritional status assessment as predictors of in-hospital mortality and readmissions in the general hospital population. Clin. Nutr..

[51] Corbacıoğlu Ş.K., Aksel G. (2023). Receiver operating characteristic curve analysis in diagnostic accuracy studies: A guide to interpreting the area under the curve value. Turk. J. Emerg. Med..

[52] Liberale L., Montecucco F., Bonaventura A., Casetta I., Seraceni S., Trentini A., Padroni M., Dallegri F., Fainardi E., Carbone F. (2017). Monocyte count at onset predicts poststroke outcomes during a 90-day follow-up. Eur. J. Clin. Investig..

[53] Zhou X., Yu F., Feng X., Wang J., Li Z., Zhan Q., Xia J. (2018). Immunity and inflammation predictors for short-term outcome of stroke in young adults. Int. J. Neurosci..

[54] DeLong J.H., Ohashi S.N., O’connor K.C., Sansing L.H. (2022). Inflammatory Responses After Ischemic Stroke. Semin. Immunopathol..

[55] Prandin G., Valente M., Zhang L., Pirera E., Malhotra P., Sacco S., Foschi M., Ornello R., Levee V., Chulack K. (2025). The impact of inflammatory markers on clinical outcomes in acute ischemic stroke patients following mechanical thrombectomy: A multicentre study. J. Neurol. Sci..

[56] Mulder I.A., van Bavel E.T., de Vries H.E., Coutinho J.M. (2021). Adjunctive cytoprotective therapies in acute ischemic stroke: A systematic review. Fluids Barriers CNS.

[57] Cao Y., Yue X., Jia M., Wang J. (2023). Neuroinflammation and anti-inflammatory therapy for ischemic stroke. Heliyon.

[58] Otani N., Hoshiyama E., Ouchi M., Takekawa H., Suzuki K. (2023). Uric acid and neurological disease: A narrative review. Front. Neurol..

[59] He W., Wu Z., Liu Y., Ye Z. (2025). Neutrophil extracellular traps in ischemic stroke: Mechanisms, clinical implications, and therapeutic potential. Front. Neurol..

